# *COL1A1* and miR-29b show lower expression levels during osteoblast differentiation of bone marrow stromal cells from Osteogenesis Imperfecta patients

**DOI:** 10.1186/1471-2350-15-45

**Published:** 2014-04-27

**Authors:** Carla M Kaneto, Patrícia SP Lima, Dalila L Zanette, Karen L Prata, João M Pina Neto, Francisco JA de Paula, Wilson A Silva

**Affiliations:** 1 Department of Genetics, Medical School of Ribeirão Preto, Universidade de São Paulo, Ribeirão Preto, São Paulo, Brazil; 2 Regional Blood Center of Ribeirão Preto and National Institute of Science and Technology in Cell Therapy, Ribeirão Preto, Brazil; 3 Department of Natural Science, Universidade Estadual do Sudoeste da Bahia, Vitória da Conquista, Bahia, Brazil; 4 Department of Clinical Medicine, Faculdade de Medicina de Ribeirão Preto, Universidade de São Paulo, Ribeirão Preto, São Paulo, Brazil; 5 Department of Biological Science, Universidade Estadual de Santa Cruz – UESC - Ilhéus, Rodovia Jorge Amado, Km16, 45662-900 Ilhéus, BA, Brazil

**Keywords:** Osteogenesis Imperfecta, miR-29b, *COL1A1*, Osteogenesis, Mesenchymalstem cells

## Abstract

**Background:**

The majority of Osteogenesis Imperfecta (OI) cases are caused by mutations in one of the two genes, *COL1A1* and *COL1A2* encoding for the two chains that trimerize to form the procollagen 1 molecule. However, alterations in gene expression and microRNAs (miRNAs) are responsible for the regulation of cell fate determination and may be evolved in OI phenotype.

**Methods:**

In this work, we analyzed the coding region and intron/exon boundaries of *COL1A1* and *COL1A2* genes by sequence analysis using an ABI PRISM 3130 automated sequencer and Big Dye Terminator Sequencing protocol. *COL1A1* and miR-29b expression were also evaluated during the osteoblastic differentiation of mesenchymal stem cell (MSC) by qRT-PCR using an ABI7500 Sequence Detection System.

**Results:**

We have identified eight novel mutations, where of four may be responsible for OI phenotype. *COL1A1* and miR-29b showed lower expression values in OI type I and type III samples. Interestingly, one type III OI sample from a patient with Bruck Syndrome showed *COL1A1* and miR-29b expressions alike those from normal samples.

**Conclusions:**

Results suggest that the miR-29b mechanism directed to regulate collagen protein accumulation during mineralization is dependent upon the amount of *COL1A1* mRNA. Taken together, results indicate that the lower levels observed in OI samples were not sufficient for the induction of miR-29b.

## Background

Osteogenesis Imperfecta (OI) (OMIM #166200) is a heterogeneous group of inherited disorders characterized by increased bone fragility and clinical severity ranging from mild to lethal [1]. Four types of OI have been described based on clinical phenotypes and histological findings [2], but more recently, at least three additional groups of patients who had a clinical diagnosis of the disorder but who presented clearly distinct features were delineated [3]. The clinical spectrum is wide, ranging from few fractures, in mild cases, to multiple fractures and perinatal lethality [4]. Most patients have mutations in the *COL1A1* or *COL1A2* genes, which encode collagen 1, which in turn is the major component of the bone matrix [5]. Collagen 1, an extra-cellular matrix protein, has two α1 and one α2 collagen chains forming a triple helix structure. However, skeletal development and bone remodeling also require stringent control of gene expression for osteoprogenitor lineage cells to advance through stages of differentiation whereas little is known about this in OI [6].

Recent reports identify critical roles of microRNAs (miRNAs) as regulators of gene expression either by inhibiting the translation or by stimulating the degradation of target mRNAs in the regulation of cell fate determination [7]. Additionally, miRNA profiling during the initial stages of osteoblast phenotype development showed the up-regulation of a limited cohort of miRNAs that have predicted targets for muscle differentiation [8]. Other works have described the importance of microRNAs in controlling transcriptions factors [9] and non-collagen extracellular matrix proteins leading to inhibition of the osteogenic process [10]. On the other hand, some microRNAs are responsible for the induction of osteogenesis [11]. MiR-29b, for example, supports osteoblast differentiation through several mechanisms which decrease the activity of *COL1A1, COL5A3* and *COL4A2* and regulate collagen protein accumulation during mineralization when miR-29b reaches peak levels [6]. There is no information regarding the expression of microRNAs in patients with Osteogenesis Imperfecta.

In this work, the analysis of *COL1A1* and *COL1A2*genes in five unrelated patients with type I and type III OI identified a total of 8 novel mutations, whereas the analysis from miR-29b expression led us to propose that it does not regulate *COL1A1* levels during osteoblast differentiation in OI in comparison to normal mesenchymal stem cells.

## Methods

The mononuclear cell fractions were derived from the bone marrow of eight different donors [five patients with Osteogenesis Imperfecta (MOOI1-MOOI5) and three normal donors (MON1-MON3)] who gave consent after full information and approval by the Ethics Committee of the Medical School of Ribeirão Preto Hospital, University of São Paulo (Number: 10188/2007). All MNC (mononuclear cells) were isolated and MSC were cultivated as previously described [12] until the third passage, when osteogenic differentiation was induced with an expansion medium supplemented with 0.01 mM dexamethasone, 200 μM ascorbic-acid-2-phosphate and 10 mM β–glycerophosphate. Genomic DNA was extracted from mesenchymal stem cells using the Wizard Genomic DNA Purification Kit (Promega). DNA sequencing of PCR-amplified *COL1A1* and *COL1A2* gene fragments covering the entire coding region and intron/exon boundaries was carried out using an ABI PRISM 3130 automated sequencer and the Big Dye Terminator Sequencing protocol. All primer sequences used in the PCR amplification of *COL1A1* and *COL1A2* are available as an Additional file 1. RNA was harvested at seven time points during the osteogenic differentiation period (D0, D + 1, D + 2, D + 7, D + 12, D + 17 and D + 21). Total RNA was isolated with TRIzol reagent (Invitrogen) and concentration was determined by photometric measurements. A High Capacity cDNA Reverse Transcription Kit (Applied Biosystems) was used to synthesize cDNA from 2 μg of RNA, following manufacturer’s recommendations. The primer sequences used in the PCR amplification of *COL1A1* are available as an Additional file 1. qRT-PCR amplification mixtures contained 20 ng template cDNA, 2X Power SYBR Green Master mix (10 μL) (Applied Biosystems) and 400–600 nM forward and reverse primers in a final volume of 20 μL. TaqMan® MicroRNA Assays (Applied Biosystems) were used to assess miR-29b expression levels and included two steps: reverse transcription and real-time PCR. The total RNA (2.5 ng/reaction) from samples was reverse-transcribed with specific looped RT primers. 15 μL reactions, in turn, were performed using reagents from the High-Capacity cDNA Archive Kit (PN 4322171, AppliedBiosystems) and 1.9 U RNase inhibitor (PNN8080119, Applied Biosystems) and were and incubated for 30 minutes at 16°C, 30 minutes at 42°C, and 5 minutes at 85°C. As for the real-time PCR step, 4.5 μL 1:5 diluted cDNA samples were used as templates in 10 μL reactions containing primers and probes for miR-29b, according to manufacturer instructions. All reactions were run in duplicate on an ABI7500 Sequence Detection System (Applied Biosystems, Foster City, CA, USA) under the following conditions: 95°C, for 10 minutes, followed by 40 cycles at 95°C for 15 seconds, and 60°C, for 1 minute. Total RNA input was normalized based on the Ct values obtained for RNU6B, which is a nucleolar RNA used as an endogenous control in this type of analysis. Experiments with coefficients of variation greater than 5% were excluded. As regards *COL1A1* reactions, each run was completed with a melting curve analysis so as to confirm the specificity of amplification and the lack of primer dimers. Reactions were carried out in triplicates and a no-template control was also included. The relative quantification of gene expression was carried out using the mathematical model described in [13].

## Results

**Figure 1:**
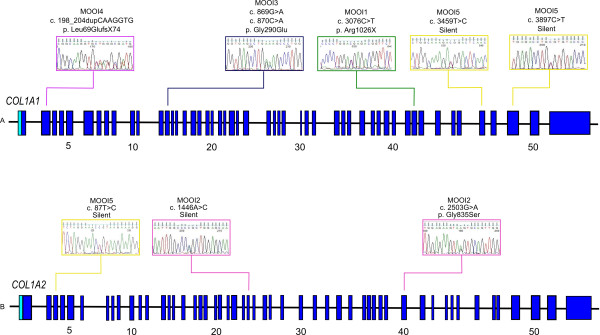
**Schematic representation of the *COL1A1 *and *COL1A2 *genes showing novel mutations described in patients with Osteogenesis Imperfecta and indicating in which exons they were found.** In *COL1A1*: 1 missense mutation (p. Gly290Glu), 1 nonsense mutation (Arg1026Ter), 1 out-of-frame insertion mutation (p. Leu69GlufsX74) and 2 silent mutations. In *COL1A2*: 1 missense mutation (p. Gly835Ser) and 2 silent mutations. The distribution of mutations in our patients is shown on Table 1.

All 51 coding exons in the *COL1A1* gene and 52 exons in the *COL1A2* gene were analyzed by DNA sequencing. A total of 8 different mutations were identified in the type I collagen genes of all patients. These mutations are summarized in Figure 1. All the mutations outlined here are novel. In *COL1A1*: 1 missense mutation (p. Gly290Glu) have been identified in patient MOOI3, 1 nonsense mutation (Arg1026Ter) have been identified in patient MOOI1, 1 out-of-frame insertion mutation (p. Leu69GlufsX74) have been identified in patient MOOI4 and 2 silent mutations were identified in patient MOOI5. In *COL1A2*: 1 missense mutation (p. Gly835Ser) have been identified in patient MOOI2 and 2 different silent mutations were identified in patient MOOI2 and MOOI5. The distribution of mutations in our patients is similar to that reported in the literature, with glycine substitutions in the helical domain, resulting in severe phenotype (Table 1). In these cases, structurally altered chains are inserted into the collagen 1 protein and disturb the formation of the triple helix, thus affecting the functions of normal chains in a secondary manner (referred to as a dominant negative effect). Nonsense mutations in *COL1A1* typically result in unstable gene products and reduced collagen synthesis, which is yet qualitatively normal. This haploinsufficiency of *COL1A1* results in mild forms of OI [14].

**Table 1:** Clinical features and detected mutations of OI patients

**Clinical data**	**MOOI-I 1**	**MOOI-III 2**	**MOOI-III 3**	**MOOI-I 4**
OI Type^*1^	I	III	III	I
Gender	M	F	M	F
Age (years)	26	23	15	41
Family history	No	No	No	No
Number of fractures	+ 30	+ 30	+ 30	− 30
Bone deformities	Yes	Yes	Yes	Yes
Blue sclera	Yes	Yes	Yes	Yes
Dentinogenesis imperfecta	Yes	Yes	Yes	Yes
Hearing loss	No	No	Yes	Yes
Mutation	COL1A1 c.3076C > T, Arg1026X	COL1A2 c.2503G > A, Gly835Ser	COL1A1 c. 869G > A, Gly290Glu	COL1A1 c.198_204dupCAAGGTG, Leu69Glu-fsX74

^*1^According to Sillence et al. [2].

**Figure 2:**
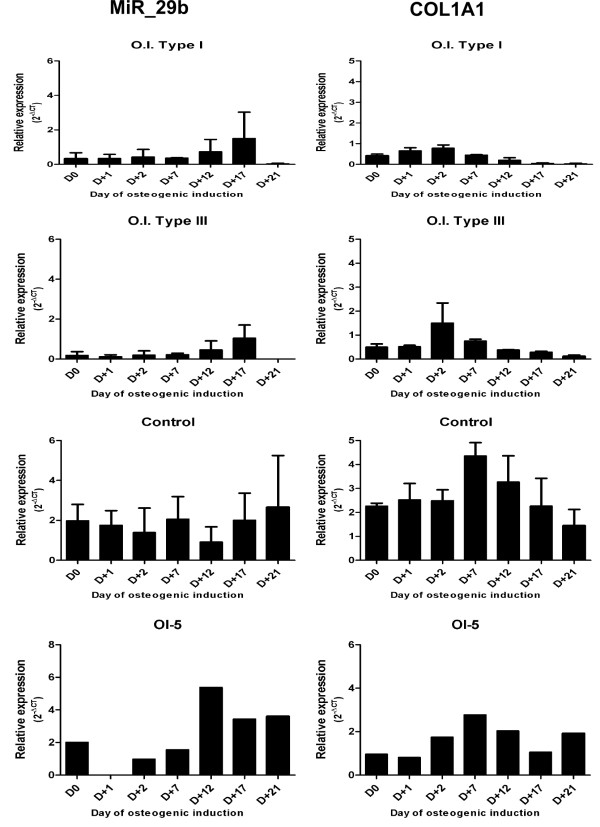
***COL1A1 *and miR-29b expression in normal, OI1 and OI3 samples during osteoblast differentiation.** Total RNA from samples of control subjects and patients was extracted and levels of miR-29b and *COL1A1* messenger RNA (mRNA) were measured quantitatively with real-time polymerase chain reaction. miR-29b and *COL1A1* expression were severely reduced in both type I and type III OI patients. The results are presented as the fold increase of expression of the individual mRNAs, with normalization with the target internal control *RNU6B* using the cycle threshold method. Data are shown as mean ± SD.

In patient OI5 with Bruck Syndrome (BS) (a very rare disorder characterized by Osteogenesis Imperfecta and arthrogryposis multiplex congenital), 3 silent mutations were detected and were considered as non-pathogenic, as they do not alter amino acids. Interestingly, miR-29b and *COL1A1* expression were severely reduced in both type I and type III OI patients (Figure 2), suggesting that miR-29b expression is not a requirement for supporting osteoblast differentiation.

## Discussion

It is possible that extracellular signals contribute to microRNA regulation during differentiation, supporting a role for microRNAs during MSC development [15]. Suh et al. (2012) have investigated the role of microRNAs in a fibroblast model of quiescence and discovered that microRNA expression is broadly and similarly altered by two different quiescence signals: contact inhibition and serum withdrawal. They further found that microRNAs regulate some of the changes in gene expression and cellular function associated with quiescence, as well as the transition between proliferation and quiescence [16]. We therefore hypothesized that the amount of mRNA and the quality of secreted collagen could be those signals. Apparently, the miR-29b mechanism for regulating collagen protein accumulation during mineralization is dependent upon the amount of *COL1A1* mRNA. The lower levels observed in OI samples are not sufficient for miR-29b induction. However, no direct interaction between *COL1A1* and miR-29b has been examined in this study.

The pattern of microRNA expression in MSC is distinct from that in pluripotent stem cells while specific populations of microRNAs are regulated in MSC during differentiation targeted towards specific cell types. Understanding the key regulatory pathways and molecules either involved in maintaining MSC in their undifferentiated state or during the process of differentiation allows for a better handle on expanding these cells for therapeutic applications on a broad scale [17].

The data obtained for BS patient revealed miR-29b and *COL1A1* expression profiles similar to those found in normal samples. Studies of Bruck syndrome have found normal secretion of collagen 1 in three families, whereas no mutations have been detected in the *COL1A1* and *COL1A2* genes [18]. In addition to the normal miR-29b and *COL1A1* expression levels, the absence of pathogenic mutations in *COL1A1* and *COL1A2* suggest that miR29b expression could be associated to *COL1A1* expression levels.

## Conclusions

Taken together**,** our study suggests that the miR-29b mechanism for regulating collagen protein accumulation during mineralization is dependent upon the amount of *COL1A1* mRNA. Results indicate that the lower levels observed in OI samples are not sufficient for the induction of miR-29b, but no direct interaction between *COL1A1* and miR-29b has been examined in this study. Furthermore, we have presented the clinical and molecular features of four patients with novel, so far undescribed mutations in *COL1A1* and *COL1A2* genes that further illustrate the complexity, but that can contribute to better understand genotype-phenotype correlations for OI and related connective tissue disorders.

## Competing interests

The authors declare that they have no competing interests.

## Authors’ contributions

CMK and PSPL performed the experiments on cell culture preparations. In the same way, CMK also conducted all experiments, differentiations, mutational analyses and gene expressions. DLZ did microRNA expression assays. KLP was responsible for collecting bone marrow samples and JMPN, FJAP and WASJ conceived of the study and participated in its design and coordination, and helped to draft the manuscript. All authors read and approved the final manuscript.

## Pre-publication history

The pre-publication history for this paper can be accessed here:

http://www.biomedcentral.com/1471-2350/15/45/prepub

## Supplementary Material

Additional file 1 Sequences of primers used for PCR, sequencing and qRT-PCR. Click here for file
