# Supplementary material for: COL1A1 and miR-29b show lower expression levels during osteoblast differentiation of bone marrow stromal cells from Osteogenesis Imperfecta patients
Source: BMC Med Genet. 2014 Apr 27;15:45. doi: 10.1186/1471-2350-15-45 (PMC4101867; doi:10.1186/1471-2350-15-45)
Supplement: Additional file 1 — Sequences of primers used for PCR, sequencing and qRT-PCR. [file 1471-2350-15-45-S1.pdf]

Additional File 1 – Sequences of primers used for PCR, sequencing and qRT-PCR.

Sequence
COL1A1-set1new-PF: CGGAGCAGACGGGAGTTTCT
COL1A1-set1new-PR: CCGAGTCTCCGGATCATCCA
COL1A1-set2new-PF: CGGGAAGTGAAAAATCCAAG
COL1A1-set2new-PR: GGAGAAGAAACAAGAGGCCA
COL1A1-set3new-PF: CTCCTGCCCTCGAATTTTGC
COL1A1-set3new-PR: GCACATGTCACAAACTGTGA
COL1A1-set4new-PF: CCAGGAAGTGCATGATGTCA
COL1A1-set4new-PR: GGTTAGAAGACAAGTCCCTG
COL1A1-set5new-PF: GGAGAGATGCTCAGAGATCT
COL1A1-set5new-PR: CCTTCCTCTGAGTATCGTTC
COL1A1-set6new-PF: CCAAAGAAGACTGAGACCTT
COL1A1-set6new-PR: GAGGTGCTTTTGGATGTCCA
COL1A1-set7new-PF: CCAAGGCTCTTTCTCAGATC
COL1A1-set7new-PR: GCTCCCATTGTCAGCCCCAA
COL1A1-set8new-PF: GGTTATGTTGGTCTGAACCC
COL1A1-set8new-PR: GTGGCACAGAGAAAGGAGTG
COL1A1-set9new-PF: CCCTTTGCCACTTTCTAACC
COL1A1-set9new-PR: GGCTCCTCTTCCTTTCTGGA
COL1A1-set10new-PF: CAGGAACCCCTGACACTGGA
COL1A1-set10new-PR: GCCTGATCCAGAACGCCTCA
COL1A1-set11new-PF: TGAGGCGTTCTGGATCAGGC
COL1A1-set11new-PR: CCAGGACTCCTTCAAGTCTC
COL1A1-set12new-PF: CCACTCAGAGTAAATGAGAG
COL1A1-set12new-PR: CCTCTAGTTGATGGCTGTCT
COL1A1-set13new-PF: GGAAGGACCGTGCTTTCCAG
COL1A1-set13new-PR: ATCTCCATGGCTTTGGTCAT
COL1A1-set14new-PF: GGAAACAAGCCTGGGAGATA
COL1A1-set14new-PR: CCAGAGAGAAGGAGAGATGC
COL1A1-set15new-PF: GCAGAGGGCCTCTCAGGAAA
COL1A1-set15new-PR: GGAGTCAGATTGGAGAGATG
COL1A1-set16new-PF: GTACAGAAGACCTGTTAAGA
COL1A1-set16new-PR: GAGAGCACAGAGGCATCAAG
COL1A1-set17new-PF: TGAGTGGCTTGGCCCTCTGT
COL1A1-set17new-PR: AGGTGCCAGAGAGCAGCACA
COL1A1-set18new-PF: GTGCCAGCTCAGATCTCTGC
COL1A1-set18new-PR: AGAGAGAGAGAAGTGAGAGT
COL1A1-set19new-PF: CCTGGTGAATCTGGACGTGA
COL1A1-set19new-PR: CCTGACATCTTGCAGGATCT
COL1A1-set20new-PF: TTGGGAGAGATGGCCACAGT
COL1A1-set20new-PR: CCATGCCCCTTCATTATTCT
COL1A1-set21new-PF: AGAATAATGAAGGGGCATGG
COL1A1-set21new-PR: GGATTACCGGCATCCAAGTG
COL1A1-set22new-PF: GGCAAAGATGGACTCAACGG
COL1A1-set22new-PR: TTGGGGTCAATCCAGTACTC
COL1A1-set23new-PF: GCCACTCTGACTGGAAGAGT
COL1A1-set23new-PR: CCCAATGCACCGTTATATCG
COL1A1-set24new-PF: CGATATAACGGTGCATTGGG

COL1A1-set24new-PR: GGAGGTCTTGGTGGTTTTGT
COL1A1-set25new-PF: GCTTCACCTACAGCGTCACT
COL1A1-set25new-PR: GGAGAAAGGAGCAGAAAGGG
COL1A1-set26new-PF: CCAAAAGTGCATTCAACCTT
COL1A1-set26new-PR: CCATCACATAGATGTAGCAC
COL1A1-set27new-PF: GGAGAGACTGTTCTGTTCCT
COL1A1-set27new-PR: GGGTCATTTCCACATGCTTT

**Sequence**
COL1A2-exon1-PF: CCTACAAGTGGCCTACAGGG
COL1A2-exon1-PF: CCTCCCATCTAACCTCTCTA
COL1A2-exon2-PF: GACCTGCATAATTTCTAGGT
COL1A2-exon2-PR: TTCATAGAAGCTGATCCTAA
COL1A2-exon3-PF: TTAGGATCAGCTTCTATGAA
COL1A2-exon3-PR: CACCAGTTTGTATCACATAA
COL1A2-exon4-PF: GCAGCTTCCAATCCTCCAGC
COL1A2-exon4-PR: GGACTGTGGTGGTAGGTAGA
COL1A2-exon5-PF: CCCTGTGATATCTTAAGAGT
COL1A2-exon5-PR: CAGTGCACACAAAGACCAGT
COL1A2-exon6-PF: GAGGTGTCGGCCAAGTTTTT
COL1A2-exon6-PR: CCCAAGTTATGGTTACTATG
COL1A2-exons7a9-PF: GGAATCAAACCACAACAATG
COL1A2-exons7a9-PR: CCATACATTAAGAGTTCTGT
COL1A2-exon10-PF: ACAGAACTCTTAATGTATGG
COL1A2-exon10-PR: TCTAAGCACAGAGTGACAAA
COL1A2-exon11-PF: TTTGTCACTCTGTGCTTAGA
COL1A2-exon11-PR: CCCTAGATAGGTCACTTAAC
COL1A2-exon12-PF: GCTGGGACCTGGAACACTGGACTTC
COL1A2-exon12-PR: TGGAGGTCATGGGGAATTTCAATCA
COL1A2-exons13a15-PF: CTGTGTGTCTGGCATAATTG
COL1A2-exons13a15-PR: AATGAAGAAGACAGCACCCA
COL1A2-exon16-PF: GTGTCATGCCACTGTAAGCA
COL1A2-exon16-PR: CTCTCTGTGGTTGACTCTGG
COL1A2-exon17-PF: CAGTAGCCAAGATGGCAGAATC
COL1A2-exon17-PR: CCAGTAAGGCCGTTTGCTCCAG
COL1A2-exon18-PF: CGTTGGACCTCCTGTAAGTAG
COL1A2-exon18-PR: AAAATGCAGTGTGGTCCATTAGG
COL1A2-exon19-PF: TAATGTGTGCTGCCTCTACAGC
COL1A2-exon19-PR: CATATAGCAGACGGGAGTGTAC
COL1A2-exon20-PF: CTTGAGCTTCTCTTTACCTTGAC
COL1A2-exon20-PR: CACCACTGGGACCAGGAGGAC
COL1A2-exon21-PF: CGTAAGTAGCTCTATCATCAC
COL1A2-exon21-PR: AAGGCAGATGGAAAGCAGATG
COL1A2-exons22a23-PF: GCAGGATGCTCATCTATGAA
COL1A2-exons22a23-PR: CTGTCAGCAAGACTACTAAC
COL1A2-exon24-PF: AAAAAGTCGGGGGAAAAGGTGCCTT
COL1A2-exon24-PR: TCTCCCCTGCTCTGCTTTCAGTCCT
COL1A2-exon25-PR: TCCCTGAGACTGGACTGATT
COL1A2-exon26-PF: CCACAGACTAGGGATCTCAA
COL1A2-exon26-PR: GCTACTACATATTCATACCC
COL1A2-exons27a28-PF: CGTGGGAACCCACAATGAGT

COL1A2-exons27a28-PR: CCAAATATCAACATGAGCAC
COL1A2-exon29-PF: GAGCTGTAAATCACCATACCGTAC
COL1A2-exon29-PR: TGGCTCATTCTCTCCATCAGCAC
COL1A2-exon30-PF: GCACTCATGTAGATACTGCC
COL1A2-exon30-PR: GGCTTTGAACATCAACACAC
COL1A2-exon31-PF: CTAGTGGAGAGATTAGGAAC
COL1A2-exon31-PR: CCACTGGAATCGGATTGCTG
COL1A2-exon32-PF: GCAGGCAAGAAGCCTGTCTA
COL1A2-exon32-PR: CCTCATGTATTACTCAACAC
COL1A2-exon33-PF: GAATGGTAAGGAATCGAGACATTGC
COL1A2-exon33-PR:
AATTTGGAAAATTCTCAATTCAACATAAAAAAAAATCCAAGTACGAAG
COL1A2-exon34-PF: CTCCTTCTGAGAGTGGCTTC
COL1A2-exon34-PR: CCTGCTGCTCTATCACAATA
COL1A2-exons35a37-PF: GTCAGTTATCTCTTCCAAGG
COL1A2-exons35a37-PR: CCTGTTGCATAGCAGGCACT
COL1A2-exon38-PF: GGTGGTAATATTGAAGAACA
COL1A2-exon38-PR: GCTGATAGCAACATACACTG
COL1A2-exon39-PF: GGTCTATTCCTGGTCACATG
COL1A2-exon39-PR: ACTTCAGACCAGGAGAGTAA
COL1A2-exon40-PF: CCAAATGGCCAGGGTATTAT
COL1A2-exon40-PR: GGCTCAACTGAGCTCTACTT
COL1A2-exon41-PF: GCCAAGATGTAAACTCACCG
COL1A2-exon41-PR: GCTGTGTCTTTATAGTGTGT
COL1A2-exon42-PF: CCTTCTTCCTTCAAACTAGA
COL1A2-exon42-PR: CCATTCTTTGGCCTAAGCAA
COL1A2-exons43a45-PF: GTGATGAAGACAGAGTAGCT
COL1A2-exons43a45-PR: CAGATGTTTTGGACTGATTC
COL1A2-exon46-PF: GTGAGAGCCTAGCTAAACCA
COL1A2-exon46-PR: GCCAGAGAATGGGAAATGGA
COL1A2-exons47a48-PF: GAGCCCCACTTTACATTTTC
COL1A2-exons47a48-PR: GAGGATATATTGAAATGGGG
COL1A2-exon49-PF: CTGATGAGAACATGCTTCCG
COL1A2-exon49-PR: CATATTTAAGAGGAAGAGGG
COL1A2-exon50-PF: CCCTCTTCCTCTTAAATATG
COL1A2-exon50-PR: CCAATCAATCCATCTTCTAA
COL1A2-exon51-PF: CCCTTTTCCTAAGCTTGGAT
COL1A2-exon51-PR: GTTAGTTCTCTCATTCTTCT
COL1A2-exon52.1-PF: GGGACAGACATCTTCAGAAT
COL1A2-exon52.1-PR: GGAAAGTGTTTTGAGGTAGT
COL1A2-exon52.2-PF: CAACACTCTTACACCTGTTA

**Sequence**
COL1A1-qRT-PF: CCTGGATGCCATCAAAGTCT
COL1A1-qRT-PF: TCTTGTCCTTGGGGTTCTTG
